# Myotonia congenita and periodic hypokalemia paralysis in a consanguineous marriage pedigree: Coexistence of a novel *CLCN1* mutation and an *SCN4A* mutation

**DOI:** 10.1371/journal.pone.0233017

**Published:** 2020-05-14

**Authors:** Chenyu Zhao, DongFang Tang, Hui Huang, Haiyan Tang, Yuan Yang, Min Yang, Yingying Luo, Huai Tao, Jianguang Tang, Xi Zhou, Xiaoliu Shi

**Affiliations:** 1 Department of Medical Genetics, The Second Xiangya Hospital, Central South University, Changsha, Hunan, China; 2 Department of Gastroenterology, The Second Xiangya Hospital, Central South University, Changsha, Hunan, China; 3 The National & Local Joint Engineering Laboratory of Animal Peptide Drug Development, College of Life Sciences, Hunan Normal University, Changsha, Hunan, China; 4 Key laboratory of Carcinogenesis and Translational Research (Ministry of Education), Intensive Care Unit, Peking University Cancer Hospital & Institute, Beijing, China; 5 Department of Rehabilitation, The Second Xiangya Hospital, Central South University, Changsha, Hunan, China; 6 Department of Neurology, The Second Xiangya Hospital, Central South University, Changsha, Hunan, China; 7 Depatment of Biochemistry and Molecular Biology, Hunan University of Chinese Medicine, Changsha, Hunan, China; NIDCR/NIH, UNITED STATES

## Abstract

Myotonia congenita and hypokalemic periodic paralysis type 2 are both rare genetic channelopathies caused by mutations in the *CLCN1* gene encoding voltage-gated chloride channel CLC-1 and the *SCN4A* gene encoding voltage-gated sodium channel Na_v_1.4. The patients with concomitant mutations in both genes manifested different unique symptoms from mutations in these genes separately. Here, we describe a patient with myotonia and periodic paralysis in a consanguineous marriage pedigree. By using whole-exome sequencing, a novel F306S variant in the *CLCN1* gene and a known R222W mutation in the *SCN4A* gene were identified in the pedigree. Patch clamp analysis revealed that the F306S mutant reduced the opening probability of CLC-1 and chloride conductance. Our study expanded the *CLCN1* mutation database. We emphasized the value of whole-exome sequencing for differential diagnosis in atypical myotonic patients.

## Introduction

Myotonia congenita (MC) is a rare genetic neuromuscular channelopathy characterized by impairment of muscle relaxation after voluntary contraction and muscle hypertrophy [1]. MC may either be autosomal recessive (Becker disease) or autosomal dominant (Thomsen disease) [2]. Both Becker disease and Thomsen disease are caused by loss-of-function mutations in the *CLCN1* gene encoding the skeletal muscle voltage-gated CLC-1 chloride channel [3]. The variants lead to hyperexcitability of the sarcolemma and resultant myotonia [4]. It was first reported in 1876 by Thomsen [5]. MC has an incidence of 1 in 100,000 individuals worldwide [6].

Since the clinical phenotypes of dystrophic myotonias (DM), sodium channel myotonia (SCM) and paramyotonia congenita (PMC) are similar, it is difficult to diagnose them by their clinical phenotypes alone. However, genetic studies have found that the occurrence of these diseases is associated with specific genetic mutations. DM results in abnormal expansion of the *DMPK* gene CTG or *ZNF9* gene CCTG repeats. SCM and PMC are caused by *SCN4A* mutations. Therefore, genetic testing plays a vital role in differential diagnosis among myotonic syndromes.

Hypokalemic periodic paralysis includes hypokalemic periodic paralysis type 1 (HypoPP1) and type 2 (HypoPP2). HypoPP2 is an autosomal dominant disorder characterized by episodic muscle weakness with hypokalemia. This is ascribed to mutations in the *SCN4A* gene encoding the α-subunit of the voltage-gated sodium channel Na_v_1.4. *SCN4A* mutations could also lead to other kinds of diseases, specifically SCM, PMC, HypoPP2 and hyperkalemic periodic paralysis (HyperPP). Mutations in HypoPP2 cause an abnormal gating pore current through the S4 segment in Na_v_1.4, which could depolarize the skeletal muscle cytomembrane, leading to weakness [7].

In this study, we describe a patient affected with both MC and HypoPP2. Whole-exome sequencing (WES) revealed that she carried two heterozygous mutations, the novel F306S in *CLCN1* and the known R222W in *SCN4A*. Sanger sequencing confirmed that her mother suffered from MC and harbored only the F306S mutation. The R222W variant was inherited from her father with HypoPP2. We investigated her complex clinical symptoms related to both diseases and the pathogenic mechanism of the F306S mutant by using the patch clamp method. We emphasized the value of WES for differential diagnosis in atypical myotonic patients and confirmed the pathogenicity of the novel F306S mutation.

## Materials and methods

### Patient information

**Fig 1:**
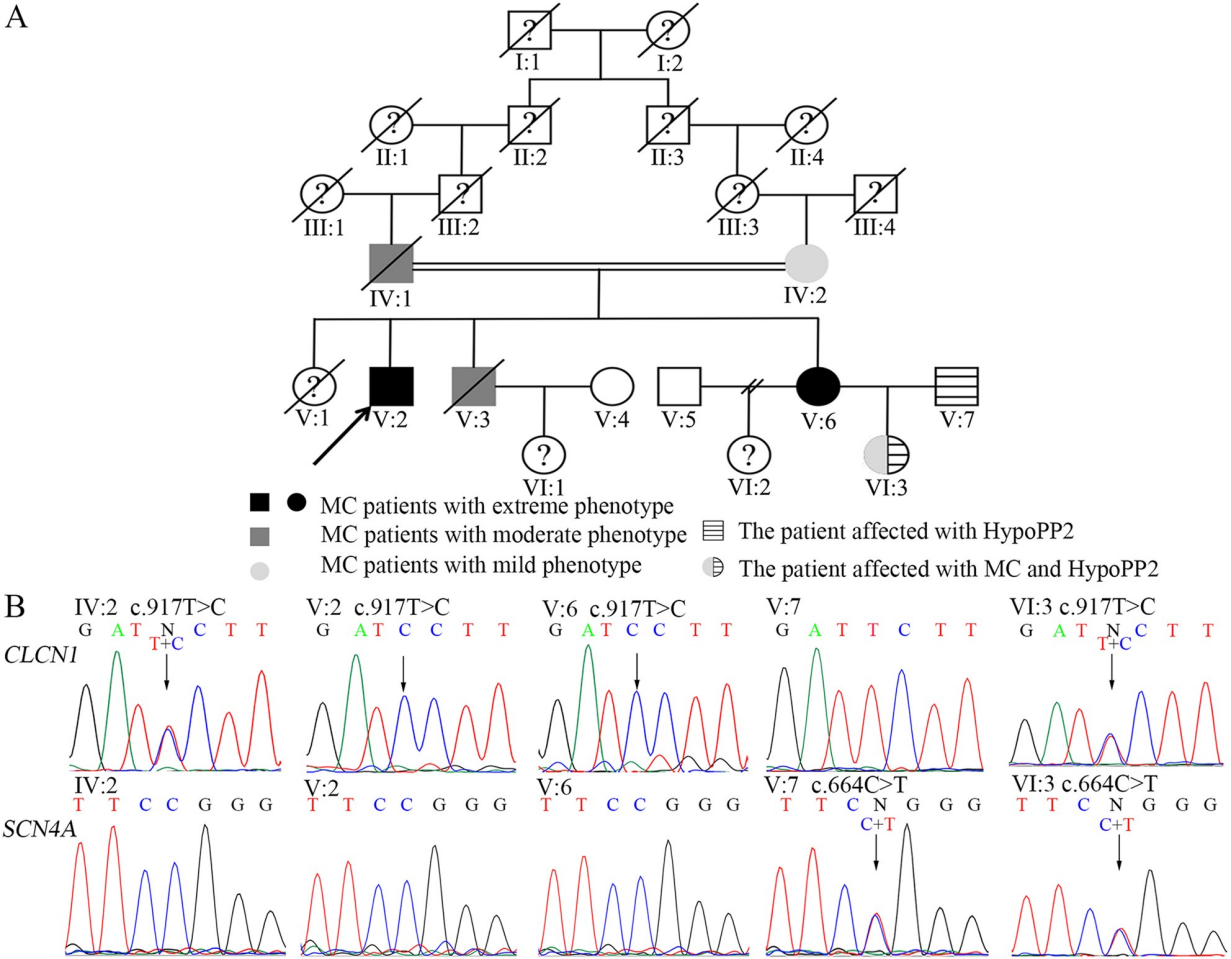
Genetic analysis. (A) The pedigree with myotonia congenital and hypokalemic periodic paralysis type 2. (B) Sanger sequencing revealed a homozygous p.F306S *CLCN1* mutation in V: 5 and V: 6, while IV: 2 and VI: 3 carried a heterozygous sequence. There was a heterozygous p.R222W *SCN4A* mutation in V.7 and VI.3.

In 2016, we enrolled a consanguineous family spanning six generations with ages ranging from 13 to 78 years from Hunan Province, China (Fig 1A). In this family, there were 5 MC sufferers (IV: 1, IV: 2, V: 2, V: 3 and V: 6). V: 7 suffered from HypoPP2. VI: 3 was affected by MC and HypoPP2. This study was approved by the ethics committee of the Second Xiangya Hospital of Central South University (2014 ethical approval No. S046). It was conducted according to the principles expressed in the Declaration of Helsinki. After receiving written informed consent, peripheral blood was collected from IV: 2, V: 2, V: 6, V: 7 and VI: 3.

### Genetic testing

Genomic DNA was isolated from peripheral blood using the QIAamp DNA Blood Mini Kit (Qiagen, Hilden, Germany).

WES was performed on V: 2, V: 6 and VI: 3. WES was performed by iGeneTech Bioscience Company Limited (Beijing, China). The exomes were captured using AIExomeV1 kits (iGeneTech Biotech, Beijing, China), and high-throughput sequencing was performed on the Solexa HiSeq2000 platform (Illumina, San Diego, USA). Basic bioinformatics analysis, including reads, mapping and variant detection, was completed by iGeneTech Bioscience Company Limited as well.

The strategies of data filtering were as follows: (1) mutations within intergenic, intronic, and untranslated regions (UTRs) and synonymous variants were excluded. (2) Variants in the database of Exome Aggregation Consortium (ExAC; http://exac.broadinstitute.org/), 1000 Genomes project (1000G; http://browser.1000genomes.org/), National Heart, Lung, and Blood Institute Exome Sequencing Project 6500 (NHLBI esp6500; http://evs.gs.washington.edu/EVS/) and Genome Aggregation (gnomAD; http://gnomad.broadinstitute.org/) with minor allele frequency>0.01 were excluded. (3) Mutations not in periodic paralysis–related and nondystrophic myotonia–related genes were excluded. There were 9 related genes, including *KCNJ2*, *KCNJ5*, *KCNJ18*, *CACNA1S*, *SCN4A*, *CLCN1*, *RYR1*, *MT-ATP6*, and *MT-ATP8* [8]. (4) Prediction software, specifically MutationTaster [9], PolyPhen-2 [10] and SIFT [11], was used to predict the effects of the variants on the protein. The pathogenicity of mutations was interpreted on the basis of the American College of Medical Genetics and Genomics (ACMG) guidance for the interpretation of sequence variants [12].

The potential variants from WES were validated by direct sequencing in the family. Primer sequences for the potential variants are provided in S1 Table. PCR products were sequenced on an ABI 3730XL Genetic Analyzer (Thermo Fisher Scientific, Inc., Waltham, MA, USA).

V: 2 and V: 6 presented with slight bilateral ankle tendon retraction. To exclude myotonic dystrophy, the length of *DMPK* gene CTG and *ZNF9* gene CCTG repeats of V: 2 were analyzed by using capillary electrophoresis.

### Functional analysis

#### Site-directed mutagenesis

The known F306L mutation, previously reported by Fialho et al. [13], and the F306S mutation were introduced into the complementary DNA of the *CLCN1* gene and subcloned into the pcDNA3.1 plasmid vector using QuikChange II XL site-directed mutagenesis (Agilent Technologies, Palo Alto, CA, USA) according to the manufacturer’s instructions. The presence of the mutations in the plasmid was verified by directed sequencing.

#### Cell culture and transfection

HEK293T cells were grown in Dulbecco’s modified Eagle’s medium supplemented with 10% fetal bovine serum, 2 mM L-glutamine, 100 U/ml penicillin and 100 μg/ml streptomycin and maintained at 37˚C with 5% CO_2_. All of the above reagents were products of Gibco^TM^ (Thermo Fisher Scientific, Inc., MA, USA). Subsequently, vectors expressing mutant and wild-type (WT) human CLC-1 channels were transfected into HEK293T cells and co-transfected with the enhanced green fluorescence protein-N1 plasmid using Lipofectamine 2000™ transfection reagent (Invitrogen; Thermo Fisher Scientific, Inc., MA, USA) according to the manufacturer’s protocol. Cells that expressed green fluorescence were used for whole-cell patch clamp analysis at 24–48 h post-transfection.

#### Electrophysiology

Whole-cell recordings of CLC-1 currents were performed using a patch clamp assay. The pipet solution (intracellular) contained 130 mM NaCl, 2 mM MgCl_2_, 5 mM EGTA and 10 mM HEPES (pH adjusted to 7.4 with NaOH). The bath solution (extracellular) contained 140 mM NaCl, 4 mM KCl, 2 mM CaCl_2_, 1 mM MgCl_2_ and 5 mM HEPES (pH adjusted to 7.4 with NaOH). All chemical reagents for the intracellular and extracellular solutions were purchased from Sigma-Aldrich (Merck KGaA, Darmstadt, Germany). Data were collected using the EPC-10 USB patch clamp platform (HEKA Elektronik, Ludwigshafen/Rhein, Germany) at room temperature (20–25˚C). Recording pipets with resistances of 2.0–3.0 MΩ were fabricated from 1.5-mm glass capillaries using a puller (PC-10; Narishige, Tokyo, Japan). An 80% series resistance compensation was used to minimize voltage errors. Voltage-dependent currents were filtered at 5 kHz and sampled at 50 kHz. Data were acquired using Patchmaster software (HEKA Elektronik).

To evoke representative current traces, cells expressing the mutant and WT human CLC-1 channels were kept at a holding potential of 0 mV, and 200-msec voltage pulses were then applied from -180 to +200 mV in 20-mV increments, followed by a -105 mV tail pulse for 200 msec every 5 sec.

**Fig 2:**
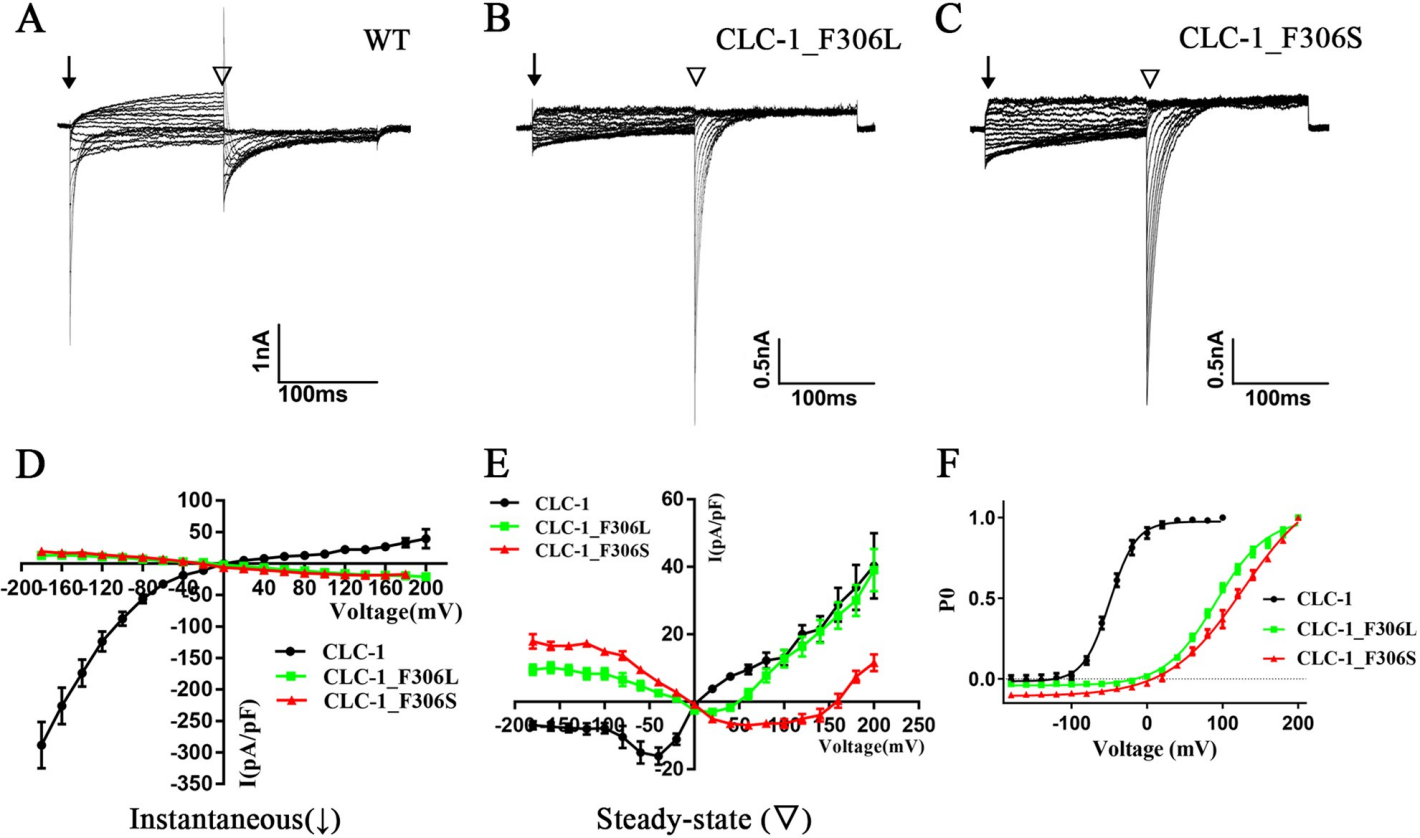
Electrophysiological properties of the CLC-1_F306S mutant channel in HEK293T cells. (A-C) Representative current traces from HEK293T cells expressing WT, CLC-1_F306L and CLC-1_F306S. (D and E) Instantaneous (denoted by an arrow) and steady-state (denoted by a triangle) current-voltage association of CLC-1_F306S and CLC-1_F306L mutant channels compared with the WT channel. (F) Voltage-dependent activation of CLC-1_F306S and CLC-1_F306L mutant channels compared with the WT channel.

Current-voltage (I-V) curves were generated by calculating the current density (I_d_) at each test voltage using the equation I_d_ = I_m_/C_m_, where I_m_ (measured in pA) and C_m_ (measured in pF) represented the transmembrane current and membrane capacitance, respectively. Steady-state and instantaneous currents were measured as indicated in Fig 2A–2C. The voltage dependence of channel activation was determined by plotting the apparent open probability (P_O_) as a function of membrane potential at the end of the test pulse. P_O_ was calculated from the peaks of the tail currents (I_v_, evoked by -105 mV) normalized to the maximal current (I_max_). Subsequently, activation curves were fitted with a Boltzmann equation: I_v_ = I_0_ + (I_max_—I_0_)/(1 + exp [(V_1/2_-V)/k]), where I_0_ is a constant offset value, V_1/2_ is the half-maximal activation voltage, V is the test voltage, and k is the slope factor.

#### Statistical analysis

Data were analyzed with Igor Pro 6.0 (WaveMetrics, Portland, OR, USA), Origin 8 (Originlab Corp., Northampton, MA, USA) and GraphPad Prism 5 (GraphPad Software, San Diego, CA, USA). Values are expressed as the mean ± standard error of the mean, and the sample size (n) represents the number of separate experimental cells. Differences between groups were analyzed using one-way ANOVA, and multiple comparisons between groups were performed by using the Tukey method with GraphPad Prism 5 (GraphPad Software, San Diego, CA, USA). P<0.05 was considered to indicate a statistically significant difference.

## Results

### Clinical findings

VI: 3 is a 12-year-old Chinese female. At nine years of age, she presented with impairment of muscle relaxation after voluntary contraction in her lower limbs. Her upper limbs were also affected by age. The myotonia could be alleviated by continued activity (warm-up phenomenon). Her clinical manifestations also include transient weakness upon initiating movements but were not associated with handgrip or lid myotonia. The myotonia symptoms did not severely impact the quality of her life. In addition, VI: 3 showed recurrent flaccid paralysis mainly involving bilateral lower limbs in 11-year-old children. The attacks always lasted for a few hours or for a day, which usually occurred after cold exposure and vigorous exercise. Periodic paralysis was not associated with dyspnea or dysarthria. Her father (V: 7) was definitively diagnosed with hypokalemic periodic paralysis in his twenties. Her mother (V: 6) suffered from myotonia and muscle hypertrophy from childhood. Physical examination revealed normal muscle force without muscle hypertrophy or myodystrophy. The percussion myotonia was negative. The laboratory tests showed no abnormalities in creatine kinase levels. Her previous examinations in another hospital suggested that the serum levels of potassium were below the normal range during paralysis attacks. Electromyography (EMG) examination revealed myotonic potential in her four limbs. Her recurrent flaccid paralysis could be relieved by oral potassium supplementation treatment. Comprehensive analysis of the clinical symptoms, family history, positive physical laboratory findings and therapeutic effects suggested a clinical diagnosis of hypokalemic periodic paralysis in combination with MC. She did not require carbamazepine treatment to alleviate her myotonia symptoms.

In the family, IV: 2, V: 2 (the proband) and V: 6 showed varying degrees of muscle stiffness and hypertrophy. Their EMG examination revealed myotonic potential. They were clinically diagnosed with MC in our hospital. IV: 1, who died a natural death, and V: 3, who died in a car accident, also had muscle stiffness and hypertrophy. We speculated that they were also affected by MC. In addition, V: 2 and V: 6 responded to carbamazepine (200 mg per day) treatment. IV: 2 did not need treatment.

Unlike other MC patients in the pedigree, the proband (V: 2), a 52-year-old Chinese male, was born from consanguineous parents (IV: 1 and IV: 2). He came to our hospital in April 2016 for the first time. EMG only identified myotonic potential. In 2019, the proband underwent EMG examination again, which showed not only myotonic potential but also slight muscle damage. Clinical findings are presented in Table 1.

**Table 1:** Summary of the family with myotonia congenital and hypokalemic periodic paralysis type 2.

Characteristics	IV: 2	V: 2 (2016)	V: 2 (2019)	V: 6	V: 7	VI: 3
Age	78	50	53	49	49	12
*CLCN1* variant	p.F306S(heterozygote)	p.F306S (homozygote)		p.F306S (homozygote)	-	p.F306S (heterozygote)
*SCN4A* variant	-	-		-	p.R222W (heterozygote)	p.R222W (heterozygote)
Lid myotonia	-	-	-	-	-	-
Handgrip myotonia	-	+	+	-	-	-
Transient weakness upon initiating movements	-	+	+	+	-	+
Minor distal weakness	-	+	+	+	-	-
Muscle stiffness	+	++++	++++	+++	-	+
Warm up phenomenon	+	++++	++++	+++	-	+
Muscle hypertrophy	-	++	++	+	-	-
Muscle force	N^a^	Level 4 in upper limbs	Level 4 in upper limbs	Level 4 in upper limbs	N	N
Slight bilateral ankle tendon retraction	-	+	+	+	-	-
Percussion myotonia	-	+	+	+	-	-
Myotonic potential in electromyography	+	+	+	+	NA^b^	+
Myogenic damage in electromyography	-	-	+	+	NA	-
Recurrent flaccid paralysis	-	-	-	-	+	+

a: normal;

b: not available

### Genetic findings

The WES yielded data covering 99.73% of the target region. Three mutations were identified during the first three steps of the filtering strategies (Table 2). Then, the pathogenicity of the mutations was predicted by MutationTaster, PolyPhen-2 and SIFT. Considering the bioinformatics analysis results and ACMG guidance for the interpretation of sequence variants, we chose the F306S *CLCN1* mutation and the R222W *SCN4A* mutation as the potential pathogenic variants.

**Table 2:** Variants identified by WES in combination with periodic paralysis–related and non-dystrophic myotonia–related gene filtering.

CHR^a^	POS^b^	RB^c^	AB^d^	Gene name	Amino acid change	MutationTaster	Polyphen-2	SIFT
17	62048561	G	A	*SCN4A*	NM_000334:exon5:c.C664T:p.R222W	Disease causing (1)	Probably damaging (1.000)	Damaging (0)
7	143027928	T	C	*CLCN1*	NM_000083:exon8:c.T917C:p.F306S	Disease causing (1)	Probably damaging (0.984)	Damaging (0)
19	38939015	G	A	*RYR1*	NM_000540:exon10:c.G821A:p.R274H	Polymorphism (0.813)	Probably damaging (0.999)	Tolerated (0.248)

a: Chromosome;

b: position;

c: reference sequence base;

d: alternative base identified

Sanger sequencing validated the two candidate variants in the pedigree (Fig 1B). VI: 3 carried both the heterozygous c.917T>C, p.F306S, g.14710T>C *CLCN1* mutation and the heterozygous c.664C>T, p.R222W, g.1718C>T *SCN4A* mutation. Nothing abnormal was detected in the length of *DMPK* gene CTG and *ZNF9* gene CCTG repeats in V: 2.

### Functional analyses

Representative current traces from HEK293T cells expressing WT, CLC-1_F306L and CLC-1_F306S are shown in Fig 2A–2C. The current values (pA) determined by the cell capacitance (pF) were used to calculate the instantaneous and steady-state current densities (pA/pF) and thereby establish I-V associations. The I-V curves of instantaneous currents for CLC-1 channels are presented in Fig 2D. At negative test voltages (ranging from -180 to 0 mV), the WT channel displayed large outward currents, while the two mutant channels (CLC-1_F306L and CLC-1_F306S) only produced a small inward rectification. In contrast, at positive test voltages (0–200 mV), the two mutant channels conducted outward currents, while the WT channel conducted inward currents. The average instantaneous current densities of mutant CLC-1_F306L and mutant CLC-1_F306S at -180 mV were 13.0±1.5 pA/pF (n = 12) and 18.6±1.6 pA/pF (n = 11), respectively. These current densities were significantly different from that of the WT channel (-288.4±36.8 pA/pF, n = 9, P<0.001). At the resting potential of normal skeletal muscle fibers (~-85 mV) [14], chloride ions were conducted outward (Cl^-^ efflux) through the WT CLC-1 channel, whereas the mutant channels facilitated a small Cl^-^ influx. Therefore, the p.F306S and p.F306L mutations in the *CLCN1* gene disrupted the chloride ion balance across the cell membrane.

The I-V curves of steady-state currents revealed that the reversal potentials of the mutant channels CLC-1_F306L and CLC-1_F306S were +50 and +160 mV, respectively and were markedly shifted in the depolarizing direction compared with the 0-mV reversal potential of the WT channel (Fig 2E).

The voltage-dependent activation curves of the WT and mutant channels, calculated from tail currents at -105 mV, are presented in Fig 2F. The V_1/2_ of the mutant channels were markedly more depolarized relative to the WT channel (F306L: 91.1±2.1 mV, n = 12; F306S: 127.0±5.9 mV, n = 11; WT: -49.2±1.8 mV, n = 9, P<0.001), with a significant alteration in the slope factors (WT: 17.4±1.6 mV, n = 9; CLC-1_F306L: 33.6±1.7 mV, n = 12; CLC-1_ F306S: 49.2±3.3 mV, n = 11, P<0.001). These results suggested that, compared with the WT CLC-1 channel, the voltage-dependent activation of two mutations at position 306 in the CLC-1 channel markedly shifted toward a positive potential.

## Discussion

VI: 3 was affected with concomitant mutations in both the *CLCN1* and *SCN4A* genes. Other researchers have also reported a few patients with the same genes mutated (Table 3) [15–18]. These patients showed an atypical phenotype, suggesting that concomitant mutations may act synergistically to influence the phenotype [19]. However, the symptoms of the two diseases so far seemed independent of each other in VI: 3. Phenotypes were not obviously different from typical HypoPP2 and MC symptoms. The possible explanation for the phenomenon varied. Because symptoms might alter or appear with age, the 12-year-old patient (VI: 3) should be followed for a more complete phenotype. Unfortunately, VI: 2, who was the elder sister of VI: 3, refused to take part in our study. As a result, we could not compare the effects of the two variants under a more similar genetic background.

**Table 3:** Coexistence of *CLCN1* and *SCN4A* mutations identified in patients.

Patient	Gender/Age	Onset age	*CLCN1* Mutation	SCN4Amutation	Phenotype	Country	Reference
1	M^a^/26	Neonatal period	p. M485V	p. G1306E	PC^c^-like phenotype. Some signs of MC^d^	France	Furby et al. [15]
2	M/13	NA	p. T268M	p. R1337P	PC-like phenotype. Some signs of MC	France	Furby et al. [15]
3	M/25	Early childhood	p. R976X	p. I693M	SCM^e^-like phenotype. Some signs of MC	France	Furby et al. [15]
4	M/27	Adolescent period	p. E950K	P. F1290L	SCM-like phenotype with periodic paralysis	Japan	Kato et al. [16]
5	F^b^/26	18	p.F167L	p.N1297S	Mild NDM^f^ phenotype. SCM-like phenotype	Italy	Maggi et al. [17]
6	M/53	Young age	p.F167L	p.N1297S	Mild NDM phenotype. SCM-like phenotype	Italy	Maggi et al. [17]
7	F/30	17	p.T550 =	p.R222Q	Severe myotonia without fulminant paralytic episodes	England	Thor et al. [18]
8	F/12	9	p.F306S	p.R222W	MC and hypoPP2	China	Zhao et al.^g^

a: Male;

b: female;

c: paramyotonia congenita;

d: myotonia congenita;

e: sodium channel myotonias;

f: nondystrophic myotonia;

g: current study

The patients’ life expectancies will not be influenced by MC. While most patients with MC do not require treatment, they may respond positively to sodium channel blockers, including mexiletine [20], phenytoin or carbamazepine [21]. Corticosteroids may also be beneficial for MC patients [22]. The homozygotes (V: 2 and V: 6) had to receive carbamazepine treatment to control severe myotonia symptoms, while the heterozygotes (IV: 2 and VI: 3) did not need that treatment. Slight atrophy may occur in some older MC patients [23]. The homozygotes (V: 2 and V: 6) manifested slight ankle tendon retraction, which may be due to a long-term lack of functional exercise. However, the older heterozygote (IV: 2) did not show the signs. Moreover, clinical findings also revealed that the homozygotes showed muscle hypertrophy, myodynamia reduction in the upper limbs, minor distal weakness, percussion myotonia, handgrip myotonia and severe muscle stiffness, which were absent or mild in heterozygous family members (Table 1). In the pedigree, the heterozygotes tended to be less severely affected than homozygotes. The patient with the heterozygous F306L mutation in another study presented with mild symptoms without muscle hypertrophy [13]. Therefore, we inferred that the rare homozygous F306S mutation might lead to an extreme phenotype.

A total of 9 CLC proteins have been identified in mammals [24]. CLC-1 is mainly expressed in skeletal muscle cell membranes [25]. CLC-1 has a double-barreled structure with one common gate exhibiting slow kinetics and two protopore gates with fast kinetics [26]. CLC-1 exists as a homodimer [27]. The mutant subunit might have a dominant-negative effect on the normal subunit [28]. Therefore, MC might be autosomal recessive (Becker disease) or autosomal dominant (Thomsen disease). Moreover, some mutations may exert a weak dominant-negative effect, resulting in reduced penetrance [29]. For example, A313T and I556N variants were found in dominant and recessive pedigrees [30]. The distribution of MC patients in the pedigree implied an autosomal dominant mode of inheritance (Thomsen disease), which was consistent with the patient with the F306L *CLCN1* mutation [13].

The F306L *CLCN1* mutation was located at the same chromosomal position as F306S but led to a different amino acid substitution. Therefore, the electrophysiological features of the WT CLC-1 channel and two mutants, F306S and F306L, were compared by heterogeneously expressing these proteins in HEK293T cells. The WT channel in skeletal muscle cells conducted an outward Cl^-^ flow to maintain the steady-state resting potential of -80 to -90 mV. The voltage-dependent activation potential of the CLC-1_F306L and CLC-1_F306S mutants was greatly shifted when compared with the WT by ~140 and ~176 mV, respectively, in the depolarizing direction. This indicated that the mutant channels were mostly closed at voltages near the resting potential and that the intracellular-to-extracellular Cl^-^ outflow was prevented in active muscle fibers. Therefore, when the skeletal muscle contracted, the muscle cells containing the *CLCN1* mutation did not repolarize to their resting potential as they normally do, and as a result, remained in an excitable state for a long period. Most pathogenic *CLCN1* mutations cause loss-of-function phenotypes in the CLC-1 channel and thus increase membrane excitability in skeletal muscle cells, consequently manifesting myotonia. The F306S mutation reduced the chloride current due to a positive shift in the voltage dependence of activation, which is consistent with one of the main dysfunction types of the *CLCN1* gene associated with MC (Table 4) [31–37].

**Table 4:** Main dysfunction types of the *CLCN1* gene associated with myotonia congenital.

Biophysical defect	Representative mutation	Reference
The voltage-dependent activation shifting toward a positive potential.	p.G190S	Desaphy et al. [31]
Positive shift of voltage dependence of slow gating	p.I290M	Pusch [32]
Inverted voltage dependence	p.D136G	Stölting et al. [33]
Decreased single channel conductance	p.C277Y	Weinberger et al. [34]
Decreased expression and decreased chloride conductance because of endosomal degradation and sarcoplasmic reticulum retention and enhanced proteasomal	p.A531V	Papponen et al.[35]
Changed outward rectification at positive potentials and halide selectivity	p.G230E	Stölting et al. [33]
Abrogated potentiation of NAD+-induced CLC-1channel inhibition	p.G200R	Bennetts et al. [36]

**Fig 3:**
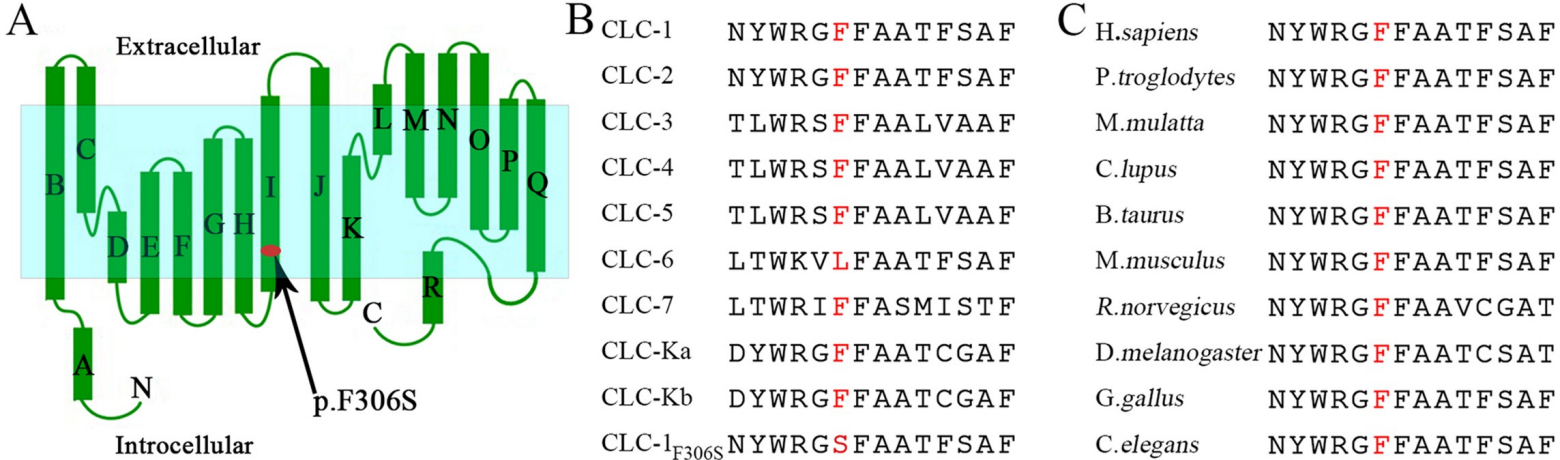
Schematic of CLC-1 channel and multiple sequence alignment. (A) The localization of p.F306S mutation on the CLC-1 channel. (B) The Phe306 amino acid residue is conserved in every member of the CLC family in humans except for CLC-6. (C) The Phe306 amino acid residue is conserved from *C*. *elegans* to *H*. *sapiens*.

To date, 321 mutations in the *CLCN1* gene have been recorded in the Human Gene Mutation Database (HGMD; http://www.hgmd.cf.ac.uk/ac/index.php). In addition, the newly discovered p.F306S mutation is a novel variant of *CLCN1*, as it has not been reported by any previous studies and is not included in the HGMD or the ExAC or the 1000G databases. A different disease-causing mutation resulting in a different amino acid substitution has already been found at this same chromosomal position [13]. The prediction software MutationTaster, PolyPhen-2 and SIFT indicated that the F306S mutation has damaging effects. Our functional experiment also supported that the novel F306S variant has a deleterious effect on CLC-1. The F306S mutation is located at helix I of the CLC-1 protein (Fig 3A). Helix I together with helix G have been reported to modulate the mechanism of the CLC-1 channel’s common gating [38]. Therefore, the F306S mutation probably has a vital role in the loss-of-function gating event. Furthermore, this phe306 amino acid residue is conserved across subtypes of human CLC proteins and species, implying that this region is functionally significant (Fig 3B and 3C). According to the ACMG guidance for the interpretation of sequence variants, we classified the mutation as a likely pathogenic variant, a term used by the ACMG to describe a variant as being disease causing with 90% certainty.

The p.R222W *SCN4A* variant has been reported in HypoPP2 patients by Matthews et al. [39]. Bayless-Edwards et al proved that the R222W variant enhanced inactivation and promoted leak currents to attenuate action potentials and depolarized muscle fibers by using patch clamp analysis [40].

Because V: 2 manifested slight ankle tendon retraction and light myogenic damage in the EMG test in 2019, we inspected dynamic mutations in V: 2. Normal length of the *DMPK* gene CTG and *ZNF9* gene CCTG repeats excluded myotonic dystrophy.

## Conclusions

In conclusion, the present study reported a patient affected with both MC and HypoPP2 in a consanguineous marriage pedigree. The novel mutation c.917T>C, p.F306S, g.14710T>C expanded the *CLCN1* gene mutation database. Furthermore, the electrophysiology data confirmed that the F306S mutation reduced the chloride conductance and opening probability of CLC-1 in skeletal muscles. Complex phenotypes led to difficulties in differential diagnosis. Therefore, we emphasized the value of WES for atypical myotonic patients.

## Supporting information

S1 Table Primer sequences used for mutation analysis of *CLCN1* and *SCN4A*. (DOCX)
