# Supplementary material for: Myotonia congenita and periodic hypokalemia paralysis in a consanguineous marriage pedigree: Coexistence of a novel CLCN1 mutation and an SCN4A mutation
Source: PLoS One. 2020 May 14;15(5):e0233017. doi: 10.1371/journal.pone.0233017 (PMC7224471; doi:10.1371/journal.pone.0233017)
Supplement: S1 Table — (DOCX) [file pone.0233017.s001.docx]

**Table S1. Primer sequences used for mutation analysis of the *CLCN1* and *SCN4A***

| Primer | Sequence (5'-3') |
| --- | --- |
| *CLCN1*-E8-F | GCACTTTCACTGCTGGCTG |
| *CLCN1*-E8-R | GTCCCACCCCACAAATGATG |
| *SCN4A*-E5-F | ATCTTTGCCATCCCCACTCC |
| *SCN4A*-E5-R | AACGCCCATCCTTCCATGTA |
